# Atypical Root Canal System Anatomy in a Permanent Upper First Molar: A Case Report

**DOI:** 10.7759/cureus.80760

**Published:** 2025-03-18

**Authors:** Slavena Georgieva, Tsvetelina Borisova-Papancheva, Denitsa Zaneva-Hristova

**Affiliations:** 1 Department of Conservative Dentistry and Oral Pathology, Medical University of Varna, Varna, BGR

**Keywords:** c-shape, endodontic treatment, maxillary first molar, root canal system configuration, root canal system variation

## Abstract

Root canal system variations can occur in each tooth group and significantly influence the outcome of the endodontic treatment. Upper first molars often present with some variations, mostly due to the presence of a second mesio-buccal root canal. Other types of atypical root canal system anatomy in upper first molars have also been reported but with a significantly smaller frequency. The aim of this article is to describe a clinical case of a C-shaped root canal configuration in a maxillary first molar - the diagnosis, preparation, irrigation, and final definitive obturation of the root canal system.

## Introduction

The main factor indicating the success of the endodontic treatment is the proper chemo-mechanical preparation and final definitive obturation of the whole root canal system. Therefore, proper knowledge of the root canal system anatomy and possible anomalies of the different tooth groups is key for the long-term success of endodontic treatment [1]. Upper first molars are one of the most investigated types of teeth that present with the most anomalies in their root canal system anatomy [2]. Different studies investigate the complex root canal system anatomy of maxillary first molars [3]. Most studies analyzing the number of roots and root canals in upper first molars conclude that they present in most cases with three roots and four root canals. A second mesio-buccal root canal is reported in 56.8% of the investigated upper first molars [4]. The presence of a fourth root canal in maxillary first molars is proven to be different in each population. The frequency is reported to be from 25% to 96.1% depending on the population investigated [5]. In the Bulgarian population, a frequency of 70.9% is being reported [6].

According to a study conducted in 2002, the presence of a C-shape configuration in maxillary first molars is 0.09% [7]. C-shaped root canal system anatomy is reported mainly in mandibular molars, although some cases of maxillary molars with this type of configuration have also been described [8]. According to Jafarzadeh and Wu, a C-shape configuration tooth has some of these features: fused roots, a longitudinal groove on the lingual or buccal surfaces of the root, and at least one cross-section of the canal that belongs to the C1, C2, or C3 configuration [9]. This anomaly of the root canal system configuration originates from the failure of Hertwig’s epithelial sheath to develop or fuse in the furcation area in the developing stage of the teeth [10].

In a 10-year study at Ghent University Hospital, only two of 2175 root-filled maxillary first molars exhibited a C-shaped configuration [7].

A different study described C-shape configuration in maxillary first molars with bilateral symmetry [11].

## Case presentation

**Figure 1:**
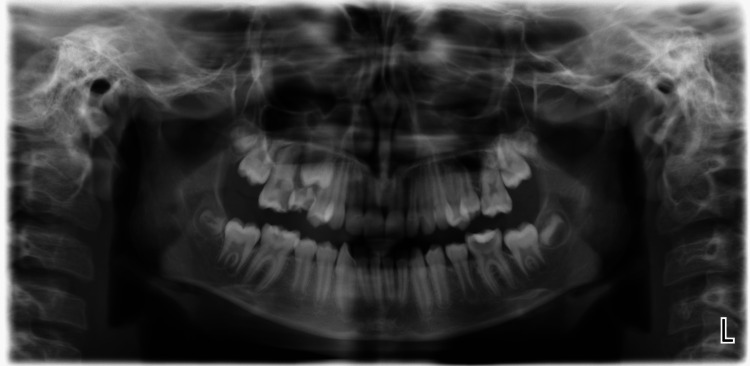
Orthopantomogram from two years ago

A 16-year-old female patient was admitted to the Medical and Dental Center at the Faculty of Dental Medicine, Medical University of Varna, Bulgaria, with complaints of irradiating pain in the upper right half of the jaw. The patient came to us with a panoramic X-ray from two years ago. It clearly shows a medial defect on tooth 16 and a rotated adjacent premolar that is erupting (Figure 1).

**Figure 2:**
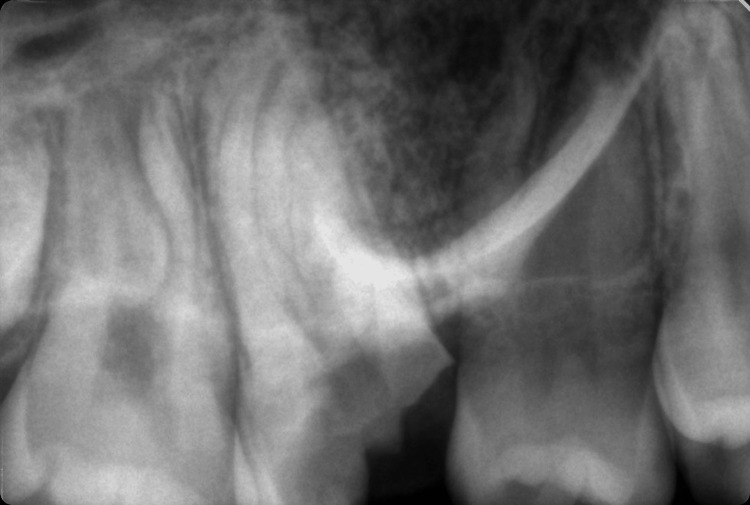
Preoperative radiographic image of tooth 16

The parents of the patient did not report any medical conditions or medication intake of their daughter. The patient was first admitted to a pediatric dentist due to her age and later transferred to a specialist in endodontics. After the radiographic examination of the patient, a large caries lesion was found on the mesioocclusal surface of tooth 16 (Figure 2). The periapical radiographic image presented an atypical root canal system anatomy for an upper first molar - three root canals were present with some connections in between them. Removal of the decayed tissues under local infiltration anesthesia with 1.7 ml 4% articaine hydrochloride Septanest 40 mg/ml 1:200.000 (Septodont, Saint-Maur-des-Fossés, France) led to pulp exposure.

After the complete removal of the decayed tissues, an endodontic pre-build-up of the mesial wall of the tooth with composite material was performed in order to achieve proper isolation of the field with a rubber dam.

Then we proceeded with the preparation of the endodontic cavity, removal of the tectum of the pulp chamber with ultrasonic tips, excavating the pulp rests from it, and finding and analyzing the floor of the pulp chamber. The floor of the pulp chamber presented with a C-shaped groove situated from mesio-buccal through disto-buccal to mesio-palatal. This finding, along with the periapical diagnostic radiograph, led to the convenience of the presence of a C-shaped root canal configuration in this upper first molar.

We proceeded with irrigation of the pulp chamber with sodium hypochlorite 5.25% (Cerkamed, Stalowa Wola, Poland) and saline and determination of the working lengths of the three root canals. For this purpose, we used small K-files Iso #10 (Dentsply Maillefer, Switzerland) and an apex locator iPex II (NSK-Nakanishi, USA). We determined the working lengths as follows: palatal root canal - 20.5 mm from the disto-palatal cusp, disto-buccal root canal - 22 mm from the buccal wall, and mesio-buccal root canal - 19 mm from the buccal wall.

The preparation of the root canals was performed with rotary files WaveOne Gold (Dentsply Sirona, Charlotte, USA), sizes small and primary. Irrigation was performed with sodium hypochlorite 5.25%, saline, and citric acid 40%, and sonic activation of the solutions with Endo Activator (Dentsply Sirona). The final irrigation sequence was sodium hypochlorite, saline, citric acid, and saline. Drying of the root canals was performed with Wave One gold paper points in the size primary.

**Figure 3:**
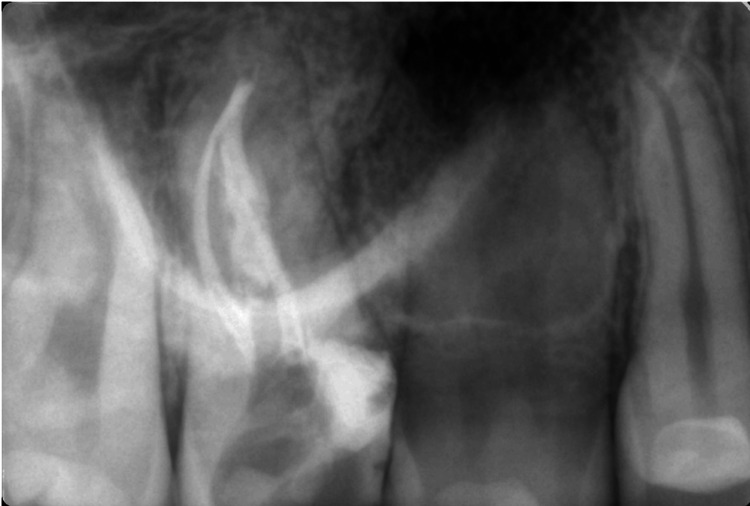
Definitive obturation of the root canals

Obturation of the root canal system was performed with gutta-percha and AH plus sealer (Dentsply Sirona) with the method of warm vertical compaction. Using this method allowed us to achieve a dense root canal system obturation (Figure 3).

The tooth was restored with a direct composite restoration. Follow-up evaluations are required to assess the long-term success of this endodontic treatment.

## Discussion

Maxillary first molars present with the greatest percentage of endodontic failure due to their complex anatomy. Different authors suggest treating this tooth always like a four-root canal tooth until proven otherwise [8]. It has been reported that only 26% of the mesio-buccal root of maxillary first molars has a single root canal [12]. Maxillary first molars with three root canals in their mesio-buccal root have also been described [13]. Clinical cases with maxillary molars with a single root canal and fused roots have also been documented [14,15]. A very limited number of C-shape configuration maxillary first molars have been described in the literature [16]. This means that although this root canal system anatomy is rare, it’s still possible to find.

In maxillary first molars, the fusion of the roots and root canals may be divided into three types: fusion of the distobuccal root with the palatal one (type A), fusion of the mesio-buccal root and the distobuccal root (type B), and fusion of two palatal roots (type C) [17].

In this article, we describe a clinical case of a C-shaped, configurated upper first molar. The coronal shape and size of the tooth crown showed no indication of this unusual anatomy, but the thorough analysis of the periapical radiographic image and later - the inspection of the pulp chamber floor with magnification proved the existence of this configuration. Using magnification enhances the chances of detecting additional root canals and more complex root canal system anatomy.

This proves that analyzing the preoperative radiographic images is as long as deep knowledge of the anatomy of the pulp chamber and the possible variations is required to perform an endodontic treatment with the best possible prognosis for the tooth.

## Conclusions

Each tooth group presents with possible anomalies of their root canal system anatomy. Maxillary first molars have a great percentage of anomalies in the number and position of the root canals and therefore present with a high percentage of endodontic failure. Each clinical case should be considered separately and a detailed analysis of the preoperative condition should be performed so that the best possible long-term prognosis of the tooth can be achieved.
